# Th.o.m.a.s.: new insights into theory of mind in adolescents with autism spectrum disorder

**DOI:** 10.3389/fpsyg.2024.1461980

**Published:** 2024-10-17

**Authors:** Roberta Fadda, Sara Congiu, Giuseppe Doneddu, Marinella Carta, Francesco Piras, Ilaria Gabbatore, Francesca M. Bosco

**Affiliations:** ^1^Department of Pedagogy, Psychology, Philosophy, University of Cagliari, Cagliari, Italy; ^2^Center for Autism and Related Disorder, Nuovo Centro Fisioterapico Sardo, Cagliari, Italy; ^3^Center for Pervasive Developmental Disorder, AO Brotzu, Cagliari, Italy; ^4^Department of Psychology, GIPSI Research Group, University of Turin, Turin, Italy; ^5^Department of Humanities, University of Turin, Turin, Italy

**Keywords:** autism spectrum disorder, theory of mind, mindreading, assessment, clinical interview, adolescents

## Abstract

Previous studies indicated atypical Theory of Mind (ToM) abilities in individuals with autism spectrum disorder (ASD) at different ages. However, research focused on adolescents with ASD is still rare. This study aims to fill the gaps in the literature, by investigating ToM abilities in adolescents with ASD and in a group of typically developing ones. We applied the Theory of Mind Assessment Scale (Th.o.m.a.s.), a semi-structured interview that allows a multi-dimensional measurement of ToM, including different perspectives (first/s-order, first/third-person, egocentric/allocentric), various mental states (emotions, desires, beliefs) and metacognitive abilities related with mental states (awareness, relation, and strategies). The results indicated that ToM develops atypically in ASD, with strengths and weaknesses. First, participants with ASD were comparable to controls in some specific ToM aspects, i.e., third-person ToM, both from an egocentric and an allocentric perspective. However, they were significantly weaker in attributing an understanding of the mental states of others, both in first- and second-order ToM scenarios. Second, they showed the same level of awareness about mental states as controls, but they were significantly weaker in conceptualizing the relationship between mental states and behavior. Also, they found it very difficult to think about possible strategies that they or others might employ to realize desires and needs. Finally, they performed similarly to controls in understanding emotions, while they poorly understood desires and beliefs. These results point out the distinctive characteristics of ToM development in individuals with ASD, with important implications for individualized interventions.

## 1 Introduction

Autism spectrum disorder (ASD) is characterized by persistent deficits in social communication and social interaction across multiple contexts. Specifically, individuals with ASD might show deficits in social–emotional reciprocity, and reduced sharing of interests, emotions, or affect with others. They might also show a deficit in nonverbal communicative behaviors used for social interaction and difficulties in developing, maintaining, and understanding relationships. Moreover, ASD is often characterized by restricted repetitive patterns of behavior (American Psychiatric Association, 2013).

The roots of the social and communicative deficit in ASD can be identified at an early age when individuals with ASD fail to develop joint attention abilities, which allow for representing the same focus of interest of another person. The lack of joint attention abilities, early in infancy, is believed to exert a cascade effect on the subsequent poor development of Theory of Mind (ToM) abilities in childhood (Mundy, 2018; Congiu et al., 2016). ToM is the ability to attribute mental states like desires, emotions, intentions, and beliefs to oneself and to others to explain and predict behavior (Wimmer and Perner, 1983). An atypical acquisition of ToM abilities is supposed to hamper social interactions in individuals with ASD during their whole lifespan (Andreou and Skrimpa, 2020; Brewer et al., 2017; Angeleri et al., 2016).

Research about ToM in ASD has mostly involved children. Recently, the interest in investigating ToM in older individuals with ASD is growing (Livingston et al., 2019). According to a recent meta-analysis (Gao et al., 2023), which considered 110 studies including 3,205 participants with ASD and 3,675 typically developing (TD) controls (mean age ≥ 18 years), indicated that late adolescents (18–24 years old) and adults with ASD demonstrate a weak performance in ToM task as compared to controls. According to Gao et al. (2023), ToM tasks that have been widely used in older individuals with ASD can be classified into four categories: *reading comprehension, perceptual scene comprehension, comprehensive scene comprehension,* and *self–other processing.*

The *reading comprehension* tasks, like for example the *Strange Stories* test (Happé, 1994), require participants to infer a character’s mental state and subsequent behavior based on the reading of relevant information in verbal vignettes.

The *perceptual scene comprehension* tasks, like for example the *animation task* (Abell et al., 2000), evaluate the ability to infer mental states behind the movement of geometrical forms (i.e., triangles) without any explicit language information and in simple social scenarios.

The *comprehensive scene comprehension* tasks, like for example *the Strange Stories Film task* (Murray et al., 2017), which consists of video scenarios based on the original *Strange Stories* (Happé, 1994): irony, double bluff, pretense, joke, appearance/reality, white-lie, persuasion, misunderstanding, forgetting, contrary emotions, and idioms. It tests the ability to attribute mental states to the characters displayed in the videos. Finally, the *self–other processing* tasks require processing a conflict between one’s own and others’ mental states and responding by shifting between one’s own and others’ points of view (Deschrijver and Palmer, 2020), which comprises explicit and implicit versions. The explicit tasks depend on language processing to stimulate individuals’ inferences about mental states, like for example the *Sandbox task* (Sommerville et al., 2013). The implicit tasks elicit rapid mental state attribution, independently from language. Implicit tasks include for example the *Reading the Mind in the Eyes test* (Baron-Cohen et al., 1997, 2001), which requires matching images of the eyes with mental state labels. Another example of implicit tasks are the eye-tracking measures of participants’ visual attention while observing an agent who holds a false belief (e.g., Senju et al., 2009).

The results of the meta-analysis (Gao et al., 2023) indicated a significant moderating effect of the ToM task’s type, since the ToM difference in *reading comprehension tasks* and *comprehensive scene comprehension tasks* was larger than that in *perceptual scene comprehension tasks* and in *self–other processing tasks*. This means that adolescents and adults with ASD might display different ToM competencies, depending on the tasks. Moreover, the ToM tasks used so far have been basically shaped by the ones originally used for young typically developing children (e.g., Baillargeon et al., 2010; Bowler, 1992; Wimmer and Perner, 1983), which tend to reduce mindreading abilities in terms of a presence/absence phenomenon (Livingston et al., 2019). Passing these kinds of tasks might not reflect the actual ToM abilities of older individuals with ASD, masking the possible difficulties that they might still experience in thinking about mental states. Indeed, ToM has a complex nature that cannot be reduced to an on–off or an all-or-nothing functioning (Tirassa et al., 2006). It is based on a developmental progression of a variety of insights about mental states like intentions, emotions, desires, knowledge, and beliefs (see, e.g., Wellman, 2014). It includes different dimensions, like the understanding of the first- and third-person perspective, which is mediated by different processes, and it recruits several types of knowledge (Nichols and Stich, 2004). It also includes the distinction between an egocentric perspective, in which the others are represented in relation to the self, and an allocentric one, in which others’ mental states are represented independently from the self (Frith and de Vignemont, 2005).

In general, previous studies using classical ToM tasks did not investigate the richness of the actual mentalization abilities in adolescent and adult individuals with ASD. This leaves open the question of whether ToM abilities of subjects in this age range who pass the classical ToM tasks are comparable to those displayed by age-matched TD controls. It is possible that other compensatory abilities, which are known to be functional to pass the classical ToM tasks, might lead individuals with ASD to interpret others’ behaviors in a very concrete and logical way, by considering external events to cause others’ behavior without the mediating effect of mental representations.

Also, it is important to consider that with aging, social-, verbal-, and nonverbal abilities tend to develop in ASD adults (Howlin and Magiati, 2017; Ratto and Mesibov, 2015). The development of these abilities might make up for the atypical ToM to a certain extent. It is possible that some adults with high-functioning ASD develop some cognitive compensation strategies that allow them to effectively perform ToM tasks thanks to their general cognitive and language skills (Frith, 1994; Begeer et al., 2010), bypassing the problem of a lack of ToM abilities. Since qualitative difficulties in social interaction persist for these individuals in everyday life, scientists assume that the use of compensatory strategies leads to passing some experimental ToM tasks (Senju et al., 2009). In line with this hypothesis, several studies indicated that linguistic and cognitive abilities, as well as executive control, significantly affect the performance of adolescents with ASD in succeeding in classical ToM tasks. A recent study explored the possible association between ToM, Executive Functioning (EF), and parent-reported measures of social communication and restricted and repetitive behaviors (RRBs) in adolescents with ASD (Jones et al., 2018). A sample of 100 adolescents with ASD (mean age 15 years 6 months) was tested by a series of ToM tasks: a false belief task, the *Strange Stories*, the Frith-Happé *animation task*, the *Reading the mind in the eyes task*. A structural equation modeling was used to verify the possible associations between ToM abilities, EF, and parent-reported measures of social communication and restricted and repetitive behaviors (RRBs). The results indicated that ToM abilities were associated with both social communication symptoms and RRBs. EF was a correlate of ToM but had no direct association with parent-reported symptom expression.

Also, according to the weak central coherence theory, adults with ASD exhibit a detail-focused style of cognition (Frith and Happé, 1994), potentially affecting information processing in ToM tasks. Furthermore, individuals with ASD might encounter difficulties in cognitive flexibility and inhibition control (Hill, 2004), which may lead to difficulties in shifting and controlling in perspective between self and others (Devine and Lecce, 2021).

In summary, previous studies indicated that individuals with ASD develop atypical ToM abilities in adolescence and adulthood. However, this research topic still deserves further investigation. On one hand, most ToM research has been focused on children, informing our understanding of mentalistic abilities and related atypical social behavior during childhood. On the other hand, only a small part of ToM research focused on adolescents and adults with ASD, therefore there is still a poor understanding of their actual mentalization abilities. Also, there are still a series of methodological concerns about the sensitivity of ToM tasks used so far. These tasks consider ToM like an all-or-nothing function, thus hampering the possibility to detect subtle distinctive features of ToM abilities in autism that might be important to develop effective intervention programs. Moreover, classical ToM tasks tap different processes underlying ToM abilities, like linguistic, cognitive abilities, and executive functioning, that might compensate for possible mindreading difficulties, leading to pass classical laboratory tasks. Finally, none of these tasks have been standardized not only in TD controls but also in other clinical populations, leaving open the question of whether some ToM deficits might be distinctive of ASD or not. These methodological concerns call for more sensitive tools to investigate mentalistic abilities in older individuals with ASD.

In this study, we investigated ToM abilities in adolescents with ASD by applying a multidimensional conceptualization of ToM abilities (Bosco et al., 2009b), compared with a group of typically developing matched controls, with the Theory of Mind Assessment Scale (Th.o.m.a.s.).

Th.o.m.a.s is a semi-structured interview that allows a multi-component and ecological measurement of different dimensions of ToM (Bosco et al., 2006): egocentric vs. allocentric perspective; beliefs vs. desires vs. positive emotions vs. negative emotions; awareness (the ability to perceive and differentiate mental states in oneself and in others) vs. causal relationships between mental states and behavior vs. efficient strategies to achieve desired states. The scale has been standardized in typical populations of adolescents and adults (Bosco et al., 2014; Bosco et al., 2016).

By adopting a multidimensional approach to investigate different ToM aspects, we aim to provide a complete, detailed, and comparable profile of mentalizing abilities in adolescents with ASD, in which specific components or sub-skills might be less or more impaired than others. We predicted that individuals with ASD, in line with other clinical populations, would show generally lower ToM abilities than controls, especially in high-level mental states like beliefs and second-order perspective.

## 2 Methods

### 2.1 Participants

We enrolled 20 participants with ASD in this study. One participant was excluded because he had a history of cognitive delay. Another one was excluded because he had a chronological age well beyond the age range of young adulthood (45 yrs). The final sample included 18 participants with ASD (3 Females; 15 Males), mean age 16 years and 5 months (± 3), and 18 typically developing controls (3 Females; 15 Males), mean age 16 years and 3 months (± 3). All participants with ASD had been diagnosed by expert clinicians and fulfilled the international diagnostic criteria of the Diagnostic and Statistical Manual of Mental Disorders 5th edition, DSM-5 (American Psychiatric Association, 2013). The diagnosis has been confirmed with the Autism Diagnostic Observation Schedule at the time of onset (Lord et al., 2005, 2013). Only 12 participants received a re-evaluation for symptom severity in adolescents according to age and verbal fluency: 8 participants were evaluated with module 4 (mean score of communication + social interaction = 6,666, SD ±3.605) while 4 were evaluated with module 3 (mean score of communication + social interaction = 7, SD ±2.966). All the participants used phrases with more than five words. Participants received education in mainstream classes in regular middle or high school. The full-scale IQ (M = 108.69, SD = 14.323, Range = 80–141) was estimated using the Wechsler Intelligence Scale for Children-Fourth Edition (WISC-IV; Wechsler, 2012). The IQ of two participants, which were, respectively, 89 and 123, was evaluated with the Wechsler Adult Intelligence Scale-Fourth Edition (WAIS-IV; Wechsler, 2013). All the subjects were recruited through the Center for Pervasive Developmental Disorder of Azienda Ospedaliera Brotzu, in Cagliari, Italy.

### 2.2 Materials and procedures

Th.o.m.a.s. is a semi-structured interview to investigate ToM (Bosco et al., 2006; Bosco et al., 2016), and has proven effective in a number of clinical populations (e.g., Bosco et al., 2009a; Laghi et al., 2014; Colle et al., 2019). It includes 37 open-ended questions that ask participants to express their understanding of their own and others’ mental states. The questions are organized into four scales: Scale A, I–Me (that investigates the interviewee’s knowledge of her own mental states—1st person ToM in an egocentric perspective); Scale B, Other–Self (which investigates the knowledge that, according to the interviewee, the other persons have of their own mental states, independently of the subject’s perspective—3rd person ToM in an allocentric perspective). Scale C, I–Other (which investigates the interviewee’s knowledge of the mental states of other persons 3rd person ToM in an egocentric perspective); Scale D, Other–Me (which investigates the knowledge that, from the interviewee’s point of view, the others have of her mental states comparable to a 2nd order ToM—in an allocentric perspective). Each scale is divided into three subscales that, respectively, explore the dimensions of Awareness (the interviewee’s ability to perceive and differentiate mental states in herself and in others), Relation (the interviewee’s ability to recognize causal relations between different mental states and between them and the resulting behaviors), and Realization (the interviewee’s ability to adopt effective strategies to achieve a desired state). The interview also allows to focus on the interviewee’s perspectives on epistemic states (knowledge, beliefs and so on), volitional states (desires, intentions, and so on), and positive and negative emotions.

To evaluate participants’ general Theory of Mind abilities, we administered a classical ToM task, consisting of a selection of four Strange Stories (Happé, 1994) to both groups of participants. Each participant was tested individually in a quiet room after signing a written consent and parents signed the consent for participants under 18 years of age. All Th.o.m.a.s. interviews were audio-recorded and then transcribed. The transcriptions were rated by two independent judges, who were blind to whether participants belonged to the experimental or the control group. Each judge was asked to evaluate each answer with a score from 0 to 4, according to the given rating criteria. To assess the inter-rater agreement an Intraclass Correlation Coefficient (ICC) was calculated on the 30% of the sample. The ICC was 0.865, indicating substantial reliability (Shrout, 1998). The study was approved by the Institutional Review Board committee of the Department of Pedagogy, Psychology, Philosophy of the University of Cagliari (Italy).

## 3 Results

Preliminary, we investigated participants’ general Theory of Mind abilities in the Strange Stories (Happé, 1994). The results indicated that participants with ASD were as able as controls in attributing mental states to the characters of the Strange Stories (*t* = 1.926; df = 33; *p* = 0.063).

To compare the performance of individuals with autism and typically developing controls on the Th.o.m.a.s. scales, we performed a repeated measures ANOVA with a two-level between-subjects factor (ASD vs. control group) and a four-level within-subjects factor (Th.o.m.a.s. scales: A (I-Me), B (Other-Self), C (I–Other), and D (Other–Me)).

The analysis revealed an effect of the group (F _(1,34) =_10.47; *p* = 0.003; η^2^_p_ = 0.235), an effect of the scale (F _(3,102)_ = 17.127; *p* < 0.001; η^2^_p_ = 0.335) and a significant Scale×Group interaction (F _(3,102)_ = 3.871; *p* = 0.011; η^2^_p_ = 0.102).

**Figure 1:**
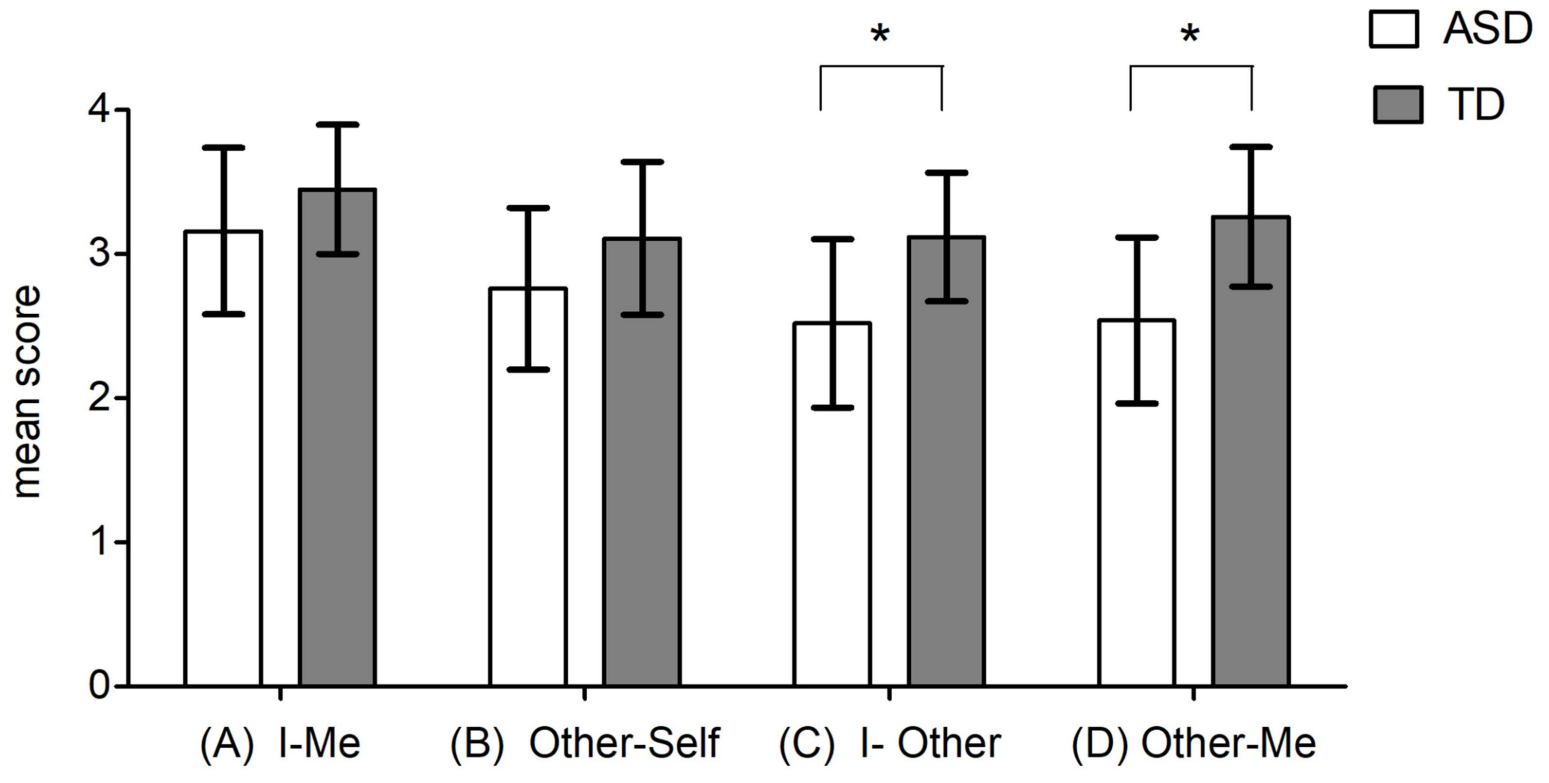
ASD vs. controls: mean scores at Th.o.m.a.s. scales: Scale A, I–Me (that investigates the interviewee’s knowledge of her own mental states—1st person ToM in an egocentric perspective); Scale B, Other–Self (which investigates the knowledge that, according to the interviewee, the other persons have of their own mental states, independently of the subject’s perspective—3rd person ToM in an allocentric perspective); Scale C, I–Other (which investigates the interviewee’s knowledge of the mental states of other persons 3rd person ToM in an egocentric perspective); Scale D, Other–Me (which investigates the knowledge that, from the interviewee’s point of view, the others have of her mental states comparable to a 2nd order ToM—in an allocentric perspective). Error bars depict a 95% confidence interval. *p < 0.012, generated by independent *t*-test.

To better explore such a result, we ran a series of t-tests (Bonferroni correction for multiple comparisons: alpha ≤0.012), which revealed that the performance of the ASD group was significantly lower than that of the control group on scale C (I-Other), investigating 3rd person ToM in an egocentric perspective (t_(34)_ = 3.462; *p* = 0.001; d = 0.599) and D (other-me), investigating egocentric second order ToM (t_(34)_ = 4.075; *p* < 0.001; d = 0.722) while no significant differences were detected in the performance of subjects with ASD and controls on scales A (I-Me), investigating first person ToM (t_(34)_ = 1.708; *p* = 0.097; d = 0.294) and B (Other-Self) investigating 3rd person ToM from an allocentric perspective (t_(34)_ = 1.923; *p* = 0.063; d = 0.350) (Figure 1).

In order to compare the performance of the two groups on Awareness, Relation, and Realization, we also run a repeated measures ANOVA with a two-level between-subjects factor (ASD vs. control group) and a three-level within-subjects factor (Th.o.m.a.s. Subscales: Awareness, Relation, and Realization). The analysis showed an effect of the group (F _(1,34)_ = 10.687; *p* = 0.002; η^2^_p_ = 0.239), an effect of the subscale (F _(2,68)_ = 3.976; *p* = 0.023; η^2^_p_ = 0.105) and a significant Group×Subscale interaction (F _(2,68)_ = 3.124; *p* = 0.05; η^2^_p_ = 0.084).

**Figure 2:**
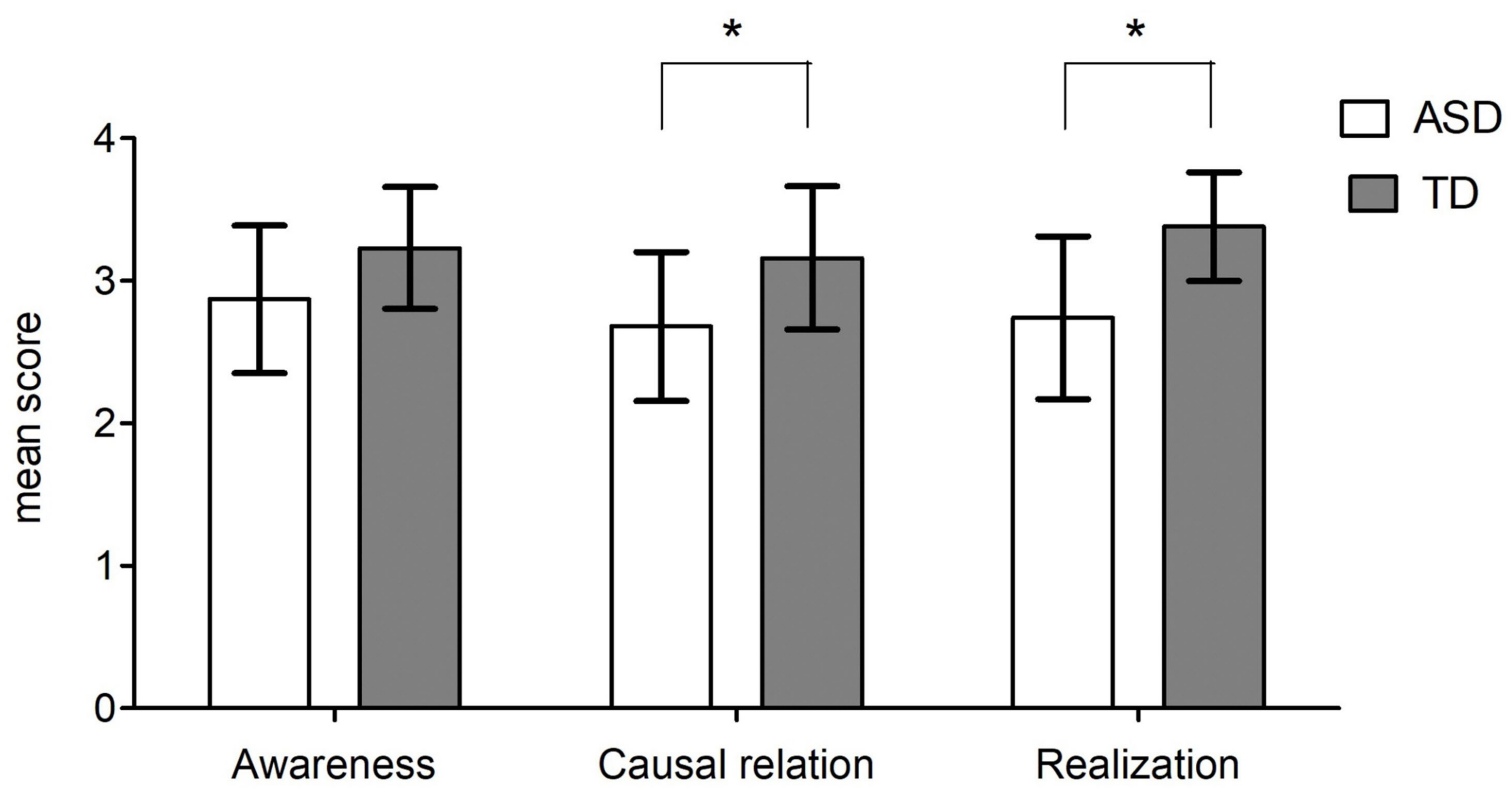
ASD vs. controls: mean scores at Th.o.m.a.s. subscales: Awareness (the interviewee’s ability to perceive and differentiate mental states in herself and in others), Relation (the interviewee’s ability to recognize causal relations between different mental states and between them and the resulting behaviors), and Realization (the interviewee’s ability to adopt effective strategies to achieve a desired state). Error bars depict a 95% confidence interval. *p < 0.017, generated by independent *t*-test.

To explore further these results, we ran a series of *t*-tests (Bonferroni correction for multiple comparisons: alpha≤0.017). As shown in Figure 2, the performance of the ASD group was significantly lower than that of the control group on the Causal Relation (t_(34)_ = 2.783; *p* = 0.009; d = 0.476) and Realization subscales (t_(34)_ = 3.948; *p* < 0.001; d = 0.639) but not on the Awareness subscale (t_(34)_ = 2.284; *p* = 0.029; d = 0.361).

**Figure 3:**
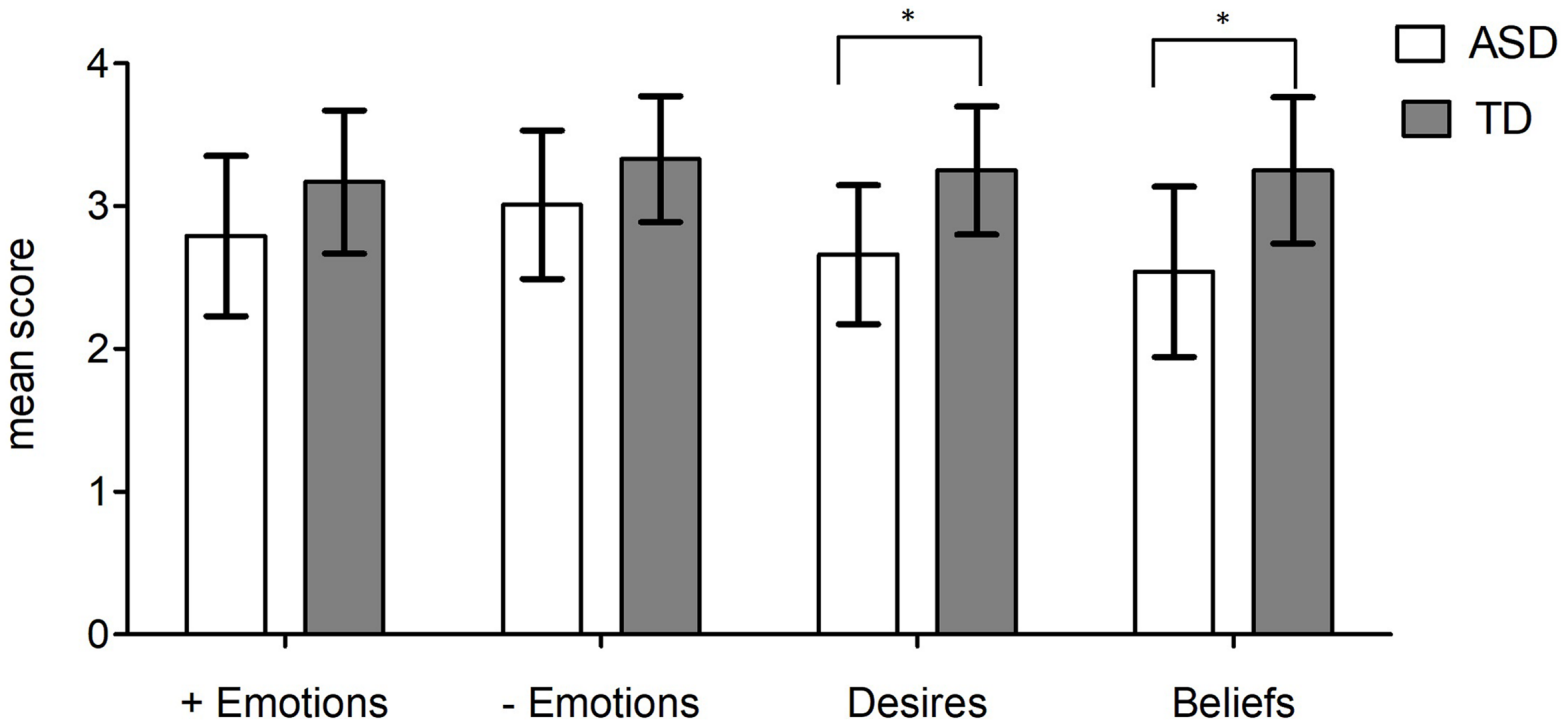
ASD vs. controls: mean scores at Th.o.m.a.s. dimensions: positive emotions (+ emotions); negative emotions (−emotions); desires; beliefs. *p < 0.017, generated by independent *t*-test. Error bars depict a 95% confidence interval.

In order to investigate the performance of the two groups in positive emotions, negative emotions, desires, and beliefs, we run a repeated measures ANOVA with a two-level between-subjects factor (ASD vs. control group) and a four-level within-subjects factor (Th.o.m.a.s. Dimensions: positive emotions, negative emotions, desires, and beliefs). The analysis revealed a significant group effect (F _(1,34)_ = 10.990; *p* = 0.002; η^2^_p_ = 0.244), a significant effect of dimension (F _(3,102)_ = 7.058; *p* < 0.001; η^2^_p_ = 0.172) and a significant Group×Dimension interaction (F _(3,102)_ = 4.029; *p* = 0.009; η^2^_p_ = 0.106). As shown in Figure 3, a series of t-tests (Bonferroni correction for multiple comparisons: alpha≤0.012) indicated that the performance of the ASD group was significantly lower than that of the control group on Desires (t_(34)_ = 3.815; *p* = 0.001; d = 0.594) and Beliefs (t_(34)_ = 3.824; *p* = 0.001; d = 0.708) but not on the Positive emotions (t_(34)_ = 2.136; *p* = 0.04; d = 0.379) and Negative emotions (t_(34)_ = 2.033; *p* = 0.05; d = 0.326).

Finally, we analyzed adolescents with ASD’s performance within-group in the four scales of the Th.o.m.a.s, by running an ANOVA, with four levels on within-subjects factors (scale type: A, I–Me; B, Other–Self; C, Me–Other; D, Other–Me). We found a significant effect of the type of scale on the ASD’s mean scores (F _(3,51)_ =13.606, *p* < 0.001, η^2^_p_ = 0.445). Specifically, *post hoc* pairwise comparison Bonferroni revealed that participants with ASD scored higher at scale A (I–Me), which assesses first-person ToM than at all the other three scales: B (Other–Self) (*p* = 0.004) and C (Me–Other) (*p* = 0.001), both of which assess third-person ToM, and D (Other–Me) (*p* < 0.001), which assesses ToM with a second-level inference. No significant differences existed between the latter three scales (Table 1).

**Table 1:** Comparison between the four scales of the Th.o.m.a.s with an ANOVA within-group in participants with autism spectrum disorder: Scale A indicates I-Me; Scale B indicates Other-Self; Scale C indicates Me-Other; Scale D indicates Other-Me.

Scale	Mean	SD	F statistics; significance *p*, դ^2^
Scale A	3.16	0.577	F _(3,51)_ = 13.606, *p* < 0.001, η^2^_p_ = 0.445
Scale B	2.76	0.562	
Scale C	2.52	0.583	
Scale D	2.54	0.576	

We also conducted a ANOVA to investigate ASD’s performance at the Th.o.m.a.s. subscales with three levels within-subjects factor (subscale type: Awareness, Relation, Realization). As it is possible to see in Table 2, there wasn’t an effect of the type of subscale and thus no difference between ASD’s mean performance (F _(1,17)_ = 2.004, *p* = 0.175, η^2^_p_ = 0.105).

**Table 2:** Comparison between the three subscales of the Th.o.m.a.s with an ANOVA within-group in participants with autism spectrum disorder: Awareness (the ability to understand self and/or other mental states), Relation (the understanding of the relationship between mental states and behavior) and Realization (the strategies that a person can use to modify self or others’ mental states).

Scale	Mean	SD	F statistics; significance *p*, դ^2^
Awareness	2.87	0.517	F _(1,17)_ = 2.004, *p* = 0.175, η^2^_p_ = 0.105
Relation	2.68	0.523	
Realization	2.74	0.571	

We investigated ASD’s performance at the four Th.o.m.a.s. dimensions (Beliefs, Desires, Positive emotions, and Negative emotions). As shown in Table 3, we found a significant effect (F _(3,51)_ = 10.250, *p* < 0.001, η^2^_p_ = 0.376). *Post hoc* pairwise comparisons revealed that participants with ASD scored higher on Negative emotions than on Positive emotions (*p* = 0.046), Desires (*p* = 1), and Beliefs (*p* = 0.078). No significant differences existed between the latter three scales.

**Table 3:** Comparison between the four dimensions of the Th.o.m.a.s with an ANOVA within-group in participants with autism spectrum disorder: Beliefs, Desires, Positive emotions, Negative emotions.

Scale	Mean	SD	F statistics; significance *p*, դ^2^
Beliefs	2.54	0.596	F _(3,51)_ = 10.250, *p* < 0.001, η^2^_p_ = 0.376
Desires	2.66	0.487	
Positive emotions	2.79	0.561	
Negative emotions	3.01	0.519	

## 4 Discussion

Our study investigated ToM abilities in adolescents with ASD with a multidimensional approach. The results indicated that ToM develops atypically in ASD, with weakness in some dimensions but not in all. First, participants with ASD scored significantly weaker than controls in the egocentric perspective. They scored also weaker than controls in the second-order understanding of mental states. However, their performance scores were comparable to those of controls when they had to reflect on mental states referred to self, from the self (egocentric perspective), and when they had to reflect on mental states that others refer to themselves (allocentric perspective).

Second, even though participants with ASD were as good as controls in the awareness of the different mental states considered in the interview, they showed significant difficulty in conceptualizing the relationship between mental states and behaviors and the possible strategies to realize desires and needs. Thus, individuals with ASD seem to be characterized by a rather descriptive Theory of Mind but not by an explanatory one. Being unable to connect different types of mental states with perceptions and actions might hamper their ability to use the knowledge about mental states to successfully affect others’ mental states. This resembles the difference between declarative and procedural knowledge about the world stored in long-term memory, which does not always match. It is possible that individuals with autism spectrum disorder might learn what a mental state actually is in a descriptive way but that they lack the possibility, for various reasons, to put into practice their knowledge. Interpreting these results in terms of cognitive models of memory, it is like if in persons with ASD the *episodic buffer* of the working memory (Baddeley, 2000) would not adequately support the memorization of the procedures needed to act adaptively in the social world. Individuals with ASD might often be more sensitive to non-social rather than to social information about the real world. The first might therefore end up being stored in their *episodic* and *semantic memory* at the expense of the second. This hypothesis is in line with the idea that ToM deficit is not the only model to explain social deficit in ASD, but that also sensory and perceptual frameworks provide an alternative explanation (e.g., Garfinkel et al., 2016).

When we considered what mental states individuals with ASD are particularly aware of, we found that participants with ASD were comparable to controls in understanding emotions, while their performance was significantly lower in understanding other mental states like desires and beliefs. Interestingly a within-group analysis revealed that they were particularly able to understand negative emotions. These results might indicate that individuals with ASD, despite well-developed linguistic and cognitive abilities, might still show a delay in the development of Theory of Mind (Yu and Wellman, 2023). Several studies in typically developing children indicated that ToM evolves with age, from infancy to childhood (see, e.g., Wellman and Liu, 2004). The understanding of emotions is the first to appear at around 2 years, followed by the understanding of desires and true beliefs at around 3 years of age, and finally false beliefs at around 4 years. It is like individuals with ASD, even though they might have a mental age equal to their peers during adolescence, are still immature in terms of their reasoning about mental states. They achieve the same level of knowledge about emotions, in particular the negative ones, which is basically the first step of the development in ToM. Individuals who interpret human behavior mainly in terms of emotions might be strongly dependent on reality, missing the constructivist activity of the mind. Thus, a certain state of the world necessarily determines specific emotions but does not elicit a desire or a belief.

ToM functioning in individuals with autism spectrum disorders shows interesting similarities and differences compared to other clinical conditions, which are characterized by significant difficulties in social relationships. In general, individuals with ASD are able to think about their own mental states from a first-order perspective, as measured in Scale A (I-Me). This is a strength also in other clinical populations, like individuals with schizophrenia, eating disorders, and borderline personality disorder (e.g., Bosco et al., 2009a; Laghi et al., 2014). However, we found that our participants with ASD show significant difficulties in recursive thinking, which is necessary to represent second-order mental states, as indicated in Scale D (Other-Me). This is in common with the other clinical conditions previously mentioned, indicating a generalized disruption of the ability to conceive the constructivist nature of other people’s minds, which can go far beyond the objective world (Bosco et al., 2009a; Laghi et al., 2014).

Although these similarities, autism spectrum disorder seems to be a peculiar condition, rather different from other clinical populations. Schizophrenia, which is a psychiatric disorder, negatively affects all the dimensions of Theory of Mind, leading to a severe misinterpretation of the social world (Bosco et al., 2009a). Mental Disorders, like eating disorders (Laghi et al., 2014) and borderline personality disorders (Colle et al., 2019), lead to hypermentalization, which is the tendency to base one’s own interpretation of social behaviors upon the content of the mind of others rather than on objective observable data. So that the interpretation of the social world might be inaccurate but only with respect to Scale B (Other-Self), which targets the allocentric third-person perspective (Colle et al., 2019).

Indeed, autism spectrum disorder, which is a long-life neurodevelopmental disorder, seems to be characterized by an inaccurate egocentric third-person perspective, as indicated by Scale C (I-Other). They consider another person’s mental states as extremely independent from themselves. Other minds are conceived as deeply opaque and highly unpredictable (i.e., “*How can I know what he feels if he does not tell me?”*). Also, they do not know how to influence others’ mental states through their behavior. Individuals with ASD do not hypermentalize. In general, they do not rely on interpersonal expectations. This means that they elaborate their interpretation of social behaviors upon their own intrapersonal expectations, which are grounded on their own state of knowledge about objective facts, as expressed in sentences like “My friend is my same age so he must hold the same desires as me, like, for example, getting a good grade at school.” Also, they base their interpretation of other minds on learned social rules and cliché, which might be a compensatory strategy to adapt to the social world. It is possible that their difficulties in representing other perspectives might lead them to hyper-generalize prototypical situations associated with a specific mental state rather than develop an effective ToM, based on their own experiences. Also, the explanation might be the other way around. It is possible that their social deficit might expose them to a major risk of isolation compared to other people. Thus, they might lack the opportunities to live the typical teenage or young adult experiences which might be fundamental to inform the development of an effective ToM. This explanation is in line with their tendency to identify their desires with the possession of items or with activities that are more typical for younger ages. Also, participants with ASD showed better performances in negative emotions compared to positive emotions, desires and beliefs. These results are in line with TD adolescents (Bosco et al., 2016) and with non-suicidal self-injury (NSSI) adolescents (Laghi et al., 2016). It seems that adolescence in general is characterized by trouble and existential confusion, which might induce individuals to be more focused on their negative emotions.

There are some possible limitations of the study that need to be acknowledged. This task requires participants to speculate on their own or others’ mental states and subsequent behavior based on memory-stored information. Thus, participants are required to retrieve prototypical information from their own experiences to infer mental states. So, information stored in long-term and working memory might play a central role. Also, this type of task also reflects linguistic skills, which are known to be related to effective ToM reasoning (Livingston and Happé, 2017). This means that our results cannot be generalized to the entire autistic spectrum. Another limitation that needs to be addressed is the reduced size of the sample. Moreover, Theory of Mind abilities might be sensitive to individual differences in symptom severity. Even though our participants were all fluent in language, received education in mainstream classes, and had the cognitive resources to attend the interview the information about the ADOS was unfortunately incomplete so we could not use it in the analysis.

As a possible future direction, we do believe that a developmental perspective might help to account for the different advances that occur in childhood and continue into adulthood in individuals with ASD. Moreover, it is important to continue to study ToM abilities in the lifespan with longitudinal studies, from adolescents to adulthood and to the elderly age, in larger samples of subjects.

## 5 Conclusion

Theory of mind is a progression of understandings about mental states, some of which may be less developed in individuals with autism at a certain point in life. Also, autism presents as a spectrum, thus some individuals with ASD achieve more theory-of-mind insights, and some achieve less. It is important to acknowledge that both theory of mind and autism are developmental phenomena, in which some advances occur in childhood and continue into adulthood. Thus, a functional and dynamic evaluation of Theory of Mind might allow us to understand that individuals with autism do not lack theory of mind overall, as a static and core characteristic. Instead, they can come up to develop many theory-of-mind competencies, although on a delayed timetable (Loukusa et al., 2023; Yu and Wellman, 2023).

Intervention in adolescents should focus mainly on second-order representation (recursive thinking) and on third-person allocentric perspective, which seems to be a long-lasting deficit in this population. Also, participation in real-life social experiences in various contexts should be recommended at this age, to promote procedural knowledge about the relationship between other high-level mental states and behavior, like beliefs and false beliefs.

## Data Availability

The raw data supporting the conclusions of this article will be made available by the authors, without undue reservation.
